# The effectiveness of electroacupuncture in the treatment of cancer-related fatigue: a systematic review

**DOI:** 10.1007/s00520-026-10651-9

**Published:** 2026-04-15

**Authors:** Maria Eduarda Alcântara Campos, Gustavo Lee Minari, Aline Teixeira Alves, Liana Barbaresco Gomide Matheus

**Affiliations:** 1 Faculty of Health Sciences and Technologies, University of Brasilia (UnB), Brasilia, Brazil; 2 Rehabilitation Sciences Program, Department of Physiotherapy, University of Brasilia (UnB), Brasília, Brazil; 3 Department of Physiotherapy, University of Brasilia (UnB), Brasilia, Brazil

**Keywords:** Electroacupuncture, Fatigue, Cancer, Oncology, Systematic review

## Abstract

**Introduction:**

Cancer-related fatigue (CRF) is a frequent, subjective, and difficult-to-manage symptom, for which few interventions have proven effective. Recent evidence suggests that electroacupuncture (EA) can reduce fatigue, although specific studies on this technique remain scarce.

**Objective:**

To evaluate the efficacy of EA in treating CRF in cancer patients.

**Methods:**

A systematic review registered in PROSPERO was conducted, including clinical trials assessing the effects of EA in patients with CRF. Searches were performed in the PubMed, Embase, Virtual Health Library, Scopus, and CAPES databases, with no restrictions on date, language, sex, ethnicity, or type of cancer. We assessed the methodological quality using the PEDro scale and evaluated the risk of bias using the RoB-2 tool.

**Results:**

A total of 2,110 studies were identified. After applying inclusion criteria, five trials were included: three randomized, one non-randomized, and one single-arm clinical trial. In total, 251 patients were analyzed, 132 of whom received EA treatment. Interventions varied from two to three weekly sessions, each lasting 20 to 30 minutes, over a period of four to eight weeks. Acupuncture points and electrical frequencies (0.2–25 Hz) differed between studies. Outcomes were measured by the Brief Fatigue Inventory, Functional Assessment of Cancer Therapy – Fatigue, and European Organization for Research and Treatment of Cancer Quality of Life Questionnaire. Despite the potential benefits observed in EA-treated groups, the findings remain inconsistent.

**Conclusion:**

EA shows promising therapeutic potential for managing CRF. However, the small number of studies, methodological heterogeneity, and small sample sizes limit generalizability. Multicenter clinical trials with larger samples and standardized protocols are needed to confirm efficacy.

## Introduction

Cancer remains one of the leading causes of mortality worldwide. According to the World Health Organization (WHO), in 2022, approximately 20 million new cases and 9.7 million deaths were reported. It is estimated that 1 in 5 individuals will develop cancer over their lifetime, which corresponds to 1 in 9 men and 1 in 12 women progressing to death. Projections indicate that these numbers will continue to increase in the coming decades, potentially surpassing 35 million new cases by 2050 [1]. Nearly one-third of cancer-related deaths are attributable to modifiable risk factors, including excess body weight, insufficient consumption of fruits and vegetables, physical inactivity, alcohol use, and tobacco smoking. Preventive strategies focused on healthy behaviors, such as adequate nutrition and regular physical activity, have substantial potential to reduce the global disease burden [2]. Although many types of cancer are now treatable, preventable, or even curable, patients frequently experience persistent mental, emotional, social, financial, and physical consequences during and after treatment [3]. Among these, cancer-related fatigue (CRF) is particularly prevalent and has a profound impact on patient’s functionality and quality of life.

CRF is defined as a distressing and subjective sensation of physical, emotional, and/or cognitive exhaustion that substantially interferes with daily functioning and is not relieved by sleep or rest [4]. It is a multifactorial condition involving several biological systems, yet it remains the symptom with the fewest evidence-based interventions, particularly when compared to others such as pain [5]. The prevalence of CRF varies, affecting approximately 40% of patients at diagnosis or after the first chemotherapy cycle, and rising to nearly 99% in advanced stages of the disease or palliative care settings [6]. Between 1996 and 2020, CRF prevalence decreased by about 20%, largely attributable to the publication of clinical guidelines for screening, assessment, and management. Nevertheless, substantial gaps in the literature persist. Although CRF has been linked to inflammatory processes, tumor lysis–related mediators, hypermetabolism, and adverse effects of antineoplastic therapies, its pathogenesis remains poorly understood, which contributes to the limited availability of effective treatments [6]. When conventional strategies are insufficient and fatigue persists, complementary and integrative approaches, such as acupuncture, may be considered.

Acupuncture, a therapeutic practice rooted in Traditional Chinese Medicine (TCM), originated in East Asia and involves the insertion of thin, sterile, disposable needles into specific anatomical points traditionally associated with symptoms or dysfunctions. Practiced for more than 2,500 years, acupuncture gained international recognition in the 1970 s [7]. In the mid-20th century, a modified technique known as electroacupuncture (EA) was developed, in which a mild electrical current is applied between pairs of acupuncture needles. Although not yet widely adopted, EA has shown comparable or even superior effects to manual acupuncture, as it minimizes dependence on practitioner skill and enables reproducibility through the use of standardized, quantifiable frequencies and intensities [8]. Emerging evidence indicates that EA may effectively reduce inflammatory cytokine levels, thereby alleviating CRF, chemotherapy-induced peripheral neuropathy, and sleep disturbances [9, 10]. Nonetheless, due to its relatively recent introduction, high-quality studies specifically investigating EA remain limited. Accordingly, this review aims to evaluate the effectiveness of EA in the management of CRF.

## Methods

This is a systematic review registered in PROSPERO (CRD420251020363), funded by the Research Support Foundation of the Federal District (FAPDF), which followed the recommendations and criteria described in the Preferred Reporting Items for Systematic Reviews and Meta-Analyses (PRISMA). The guiding question of this systematic review was: "Is electroacupuncture effective in the treatment of cancer-related fatigue?" To construct this question, the PICOT strategy was used: P as the population (cancer patients), I as the intervention (electroacupuncture), C for comparison (any control group, placebo, or compared with the intervention), O as the outcome (fatigue assessment instruments), and T as the time (during or after oncological treatment).

### Search Strategy

The search was conducted on July 24, 2025 in five databases: National Library of Medicine (Medline® via PubMed®), Embase, Virtual Health Library (BVS), Scopus, and CAPES journals. Controlled descriptors indexed in Medical Subject Headings (MeSH) and their synonyms in Health Science Descriptors (DeCS) were carefully selected to broaden the search and address the research question using PICOT. Keywords such as “electroacupuncture,” “neoplasm,” “cancer,” “fatigue,” “cancer-related-fatigue,” and “oncology” were employed with Boolean operators OR and AND. Detailed strategies are summarized in Table 1. After the database search, the studies were imported into Rayyan, a free software developed by the Qatar Computing Research Institute (QCRI), where the initial screening of articles and duplicate removal were performed. Article selection occurred in three stages: reading of titles, abstracts, and full texts, conducted independently by two reviewers. Discrepancies were resolved by a third researcher.

**Table 1:** Detailed search strategy

DATABASES	SEARCH STRATEGY
Pubmed (MeSH) n = 82	((Electroacupuncture OR electric acupuncture OR electrical acupuncture OR electro-acupuncture OR electronic acupuncture) AND (fatigue)) AND (Neoplasms OR Neoplasm OR Tumors OR Neoplasia OR Neoplasias OR Tumor OR Cancer OR Cancers OR "Malignant Neoplasm" OR Malignancy OR Malignancies OR "Malignant Neoplasms" OR Oncology)
Embase (Emtree) n = 89	('Electroacupuncture'/exp OR 'Electroacupuncture' OR 'acupuncture, electric' OR 'electric acupuncture' OR 'electrical acupuncture' OR 'electro-acupuncture' OR 'electrode acupuncture' OR 'electronic acupuncture') AND ('cancer fatigue'/exp OR 'cancer fatigue' OR 'fatigue') AND ('Malignant Neoplasms'/exp OR 'Malignant Neoplasms' OR Neoplasms OR Neoplasm OR Tumors OR Neoplasia OR Neoplasias OR Tumor OR Cancer OR Cancers OR Oncology)
SCOPUS n = 1,860	(ALL ('electroacupuncture' OR 'electro-acupuncture' OR "electrode acupuncture" OR "electronic acupuncture") AND ALL ('fatigue' OR "cancer fatigue" OR 'cancer-related-fatigue') AND ALL (neoplasms OR neoplasm OR tumors OR neoplasia OR neoplasias OR tumor OR cancer OR cancers OR "Malignant Neoplasm" OR malignancy OR malignancies OR "Malignant Neoplasms" OR oncology))
CAPES n = 29	"Electroacupuncture" AND "fatigue" AND "cancer"
BVS n = 24	(electroacupuncture OR "electric acupuncture" OR "electrical acupuncture" OR electro-acupuncture OR "electrode acupuncture" OR "electronic acupuncture") AND (fatigue OR "cancer, fatigue") AND (neoplasms OR neoplasm OR tumors OR neoplasia OR neoplasias OR tumor OR cancer OR cancers OR "Malignant Neoplasm" OR malignancy OR malignancies OR "Malignant Neoplasms" OR oncology) AND instance:"regional"

### Selection criteria

Randomized and non-randomized clinical trials investigating the effects of EA in patients with CRF were included. Case reports, literature reviews, study protocols, observational studies, conference posters, and dissertations were excluded. Studies that combined EA with other interventions or employed solely other techniques, that involved patients with conditions other than cancer, or that assessed only outcomes unrelated to fatigue were also excluded.

### Study screening

After removing duplicates, screening was conducted in two stages by two authors: initial reading of titles and abstracts, followed by full-text reading. Data were extracted into a standardized Excel spreadsheet: author, year, title, study type, sample size, cancer type, groups, duration (weeks), frequency, assessment tool, intervention and results.

### Assessment of methodological quality and risk of bias

The methodological quality of the included clinical trials was determined using the PEDro scale. The scale consists of 11 criteria: specified eligibility, random allocation, concealed allocation, baseline comparability, participant blinding, therapist blinding, assessor blinding, adequate follow-up, intention-to-treat analysis, between-group comparisons, and measures of precision and variability. Each item is scored “yes” or “no.” Because criterion 1 is not used in the calculation, the maximum PEDro score is 10 points. Trials scoring PEDro ≥ 6 points were considered methodologically high quality, while trials scoring PEDro < 6 were classified as low quality. To analyze the Risk of Bias of the included randomized clinical trials, the Cochrane Risk of Bias tool (ROB-2) was used. The tool comprises five domains: randomization bias, bias due to deviations from intended interventions, bias due to missing outcome data, bias in outcome measurement, and bias in selection of the reported results. Each domain is evaluated through questions with five possible answers: “yes”, “probably yes”, “no”, “probably no”, and “not reported”. Each domain is ultimately rated as low risk, some concerns, or high risk of bias. All assessments were independently conducted by two researchers, with discrepancies resolved by a third reviewer.

## Results

### Literature search and study selection

**Fig. 1:**
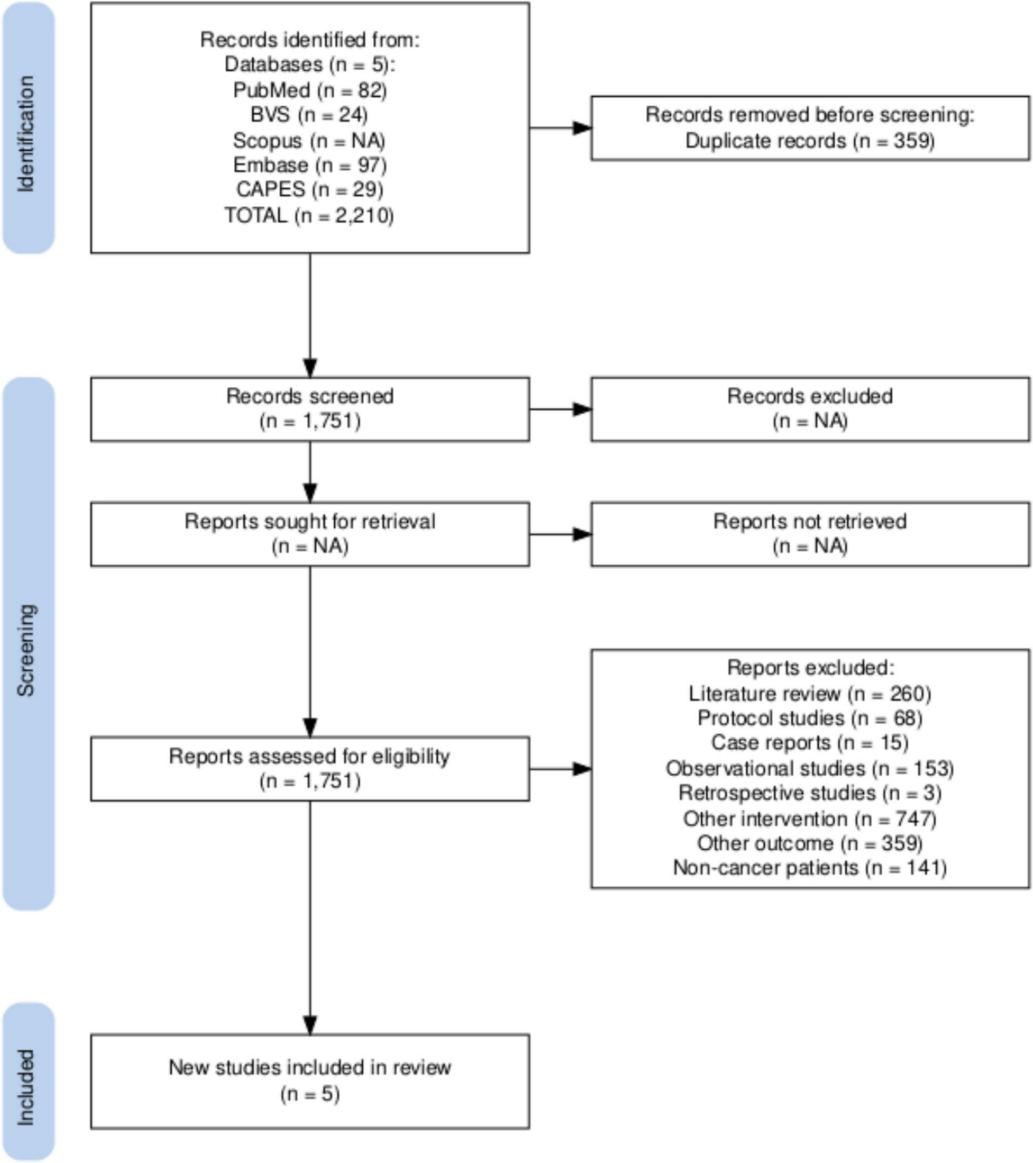
PRISMA Flowchart [18]

A total of 2,110 studies were found and imported into Rayyan. Following the removal of 359 duplicates, 1,751 studies remained for screening. In the initial evaluation by title, 1,246 studies were excluded, comprising 242 systematic reviews, 18 integrative literature reviews, 68 study protocols, 15 case reports, 153 observational studies, 3 retrospective studies, and 747 studies that employed other techniques (such as acupuncture, acupressure, moxibustion, aromatherapy, among others) and/or included additional interventions within the same control group. The remaining 500 studies were assessed based on their abstracts, of which 359 were excluded for not evaluating fatigue as an outcome and 141 for including patients with conditions other than cancer. Ultimately, three randomized clinical trials were included. Given the scarcity of studies in this field, one single-arm clinical trial and one non-randomized clinical trial were also incorporated (see PRISMA flowchart, figure 1).

### Characteristics of included studies

The oldest clinical trial identified was conducted by Mao et al., 2009, in a single-arm study that investigated the effects of EA on fatigue in women with breast cancer using aromatase inhibitors [19]. Subsequently, in 2014, the same author published a randomized clinical trial conducted at the Abramson Cancer Center at the University of Pennsylvania Hospital (Philadelphia, USA), which was also included in this review [20]. More recently, randomized clinical trials by Saraswati et al., 2021 and Lee et al., 2022 were identified. These studies differ from the previous ones in that they include patients with other types of cancer, such as cervical, thyroid, and gastrointestinal cancer, whereas all other studies were in women with a history of breast cancer [21, 22]. The most recent study we selected was that of Zhao et al., published in 2023, which, even though it was a non-randomized clinical trial, sought to answer the hypothesis that single nucleotide polymorphisms can be markers used to accurately assess the effectiveness of electroacupuncture as a treatment for fatigue in breast cancer patients after chemotherapy [23]. Methodological characteristics and results are detailed in Table 2.

**Table 2:** Characteristics of the included studies

Author/ year	Title	Study type	Sample (n = total)	Cancer type	Groups	Duration (weeks)	Frequency	Outcomes	Assessment tool	Intervention	Results
Mao et al., 2009	Feasibility Trial of Electro- acupuncture for Aromatase Inhibitor Related Arthralgia in Breast Cancer Survivors	SCT	12	Breast cancer	EA	8	Twice a week for two weeks, then weekly for another six weeks	MO: Pain; Functional interference SO: Fatigue, Anxiety and depression; Sleep disturbance	Brief Fatigue Inventory (BFI)	EA group: Needles were inserted until “De Qi” was reported by patients. Two pairs of electrodes were connected with 2 Hz electrostimulation provided by a TENS unit for 20 min	The subjects showed a significant reduction in fatigue from 4.38 to 1.93 (p = 0.005)
Mao et al., 2014	Electroacupuncture for Fatigue, Sleep, and Psychological Distress in Breast Cancer Patients With Aromatase Inhibitor-Related Arthralgia: A Randomized Trial	RCT	67	Breast cancer	EA; SEA and Control	8	Twice a week for two weeks, then weekly for another six weeks	MO: Pain intensity and interference SO: Fatigue, Sleep; Psychological distress	Brief Fatigue Inventory (BFI)	EA group: Needles were inserted until the patient reported “De Qi.” Two pairs of electrodes were connected with 2 Hz supplied by TENS for 30 min SEA Group: Non-penetrating needles were used on non-acupuncture and non-trigger points. Acupuncturists avoided provoking “De Qi” sensations and connected the TENS unit to a different channel so that the participant could observe the light without receiving electricity	The EA group showed significant improvement compared to the control group (p = 0.0095), while the SEA group showed no improvement (p = 0.18)
Saraswati et al., 2021	The Effect of Electroacupuncture Therapy on Pain, Plasma β-Endorphin, and Quality of Life of Stage III Cervical Cancer Patients: A Randomized Control Trial	RCT	28	Cervical cancer	EA and Control	3	10 sessions every 2 days	MO: Pain; Plasma β-endorphins levels; Quality of Life SO: Fatigue; Sleep; Appetite; Nausea, vomiting; Congestion; Constipation, diarrhea; Finance	European Organization for Research and Treatment of Cancer Quality of Life Questionnaire (EORTC QLQ-C30)	EA group: needles were placed at LI4, ST36, SP6, and LR3 on symmetrical parts of the right and left sides of the body and then connected to the electroacupuncture device at 2/20–25 Hz for 30 min	There was a significant decrease in fatigue in the EA group (p = 0.001) and control group (p = 0.038)
Lee et al., 2022	Electroacupuncture for treating cancer-related insomnia: a multicenter, assessor-blinded, randomized controlled, pilot clinical trial	RCT	20	Breast cancer, thyroid, and gastro- intestinal cancer	EA; SEA and Control	4	2–3 times a week	MO: Insomnia SO: Fatigue; Cognitive Assessment; Levels of salivary cortisol and melatonin	Functional Assessment of Cancer Therapy- Fatigue (FACT-F)	EA group: needles at GV20, EX-HN3, HT7, PC6, BL63, and KI4. After achieving the sensation of “de qi” by rotating the needles, a device was used at a frequency of 4 Hz for 30 min SEA group: the placebo acupuncture needle, which did not penetrate the skin, was inserted into 10 non-acupuncture points. The deactivated EA device was connected, emitting the same sound and lights without providing any electrostimulation	The EA group showed significant improvement compared to the SEA group 4 weeks after treatment (p = 0.0305)
Zhao et al., 2023	Screening Single Nucleotide Polymorphisms Predicting the Efficacy of Electroacupuncture for Fatigue Treatment in Patients with Breast Cancer Following Adjuvant Chemotherapy	NRCT	124	Breast cancer	EA and Control	S/I	Each patient received treatment within 6 h after chemo- therapy in the second cycle, and were treated every 2 days for six cycles	MO: Fatigue; Single Nucleotide Polymor- phisms	Brief Fatigue Inventory (BFI)	EA group: needles at ST36, SP6, and LI4 After achieving the sensation of “de qi,” the needle was electrified for 30 min, with a continuous wave type, frequency of 0.2 Hz, and current intensity ≤ 10 mA	The fatigue symptoms of 26.9% of patients improved significantly (p < 0.05), and there was a statistical difference between the EA group and the control group (p = 0.037)

**Legend:** SCT—single-arm clinical trial; RCT—randomized clinical trial; NCT—non-randomized clinical trial; EA—electroacupuncture; SEA—simulated electroacupuncture; MO—Main Outcomes; SO—Secondary Outcomes

### Assessment tool

Three of the five studies measured the outcome using the Brief Fatigue Inventory (BFI) score, while Lee et al., 2022 and Saraswati et al., 2021 evaluated it using the Functional Assessment of Cancer Therapy - Fatigue (FACT-F) and European Organization for Research and Treatment of Cancer Quality of Life Questionnaire (EORTC QLQ-C30), respectively. The BFI is a nine-item instrument rated on a numerical scale from 0 to 10, in which patients indicate the intensity of their fatigue at different time points and assess the extent to which fatigue has interfered with various aspects of their lives over the previous 24 hours ​[11]​. The FACT-F evaluates five domains: physical well-being, social/family well-being, emotional well-being, functional well-being, and fatigue. Each item is scored on a five-point Likert scale ranging from 0 to 4. The final FACT-F score is obtained by summing the five domains and ranges from 0 to 160 points. Higher scores indicate better quality of life and lower levels of reported fatigue ​[12]​. The EORTC QLQ-C30 consists of nine multidimensional scales: five functional scales, one global health status/quality of life scale, and three symptom scales (fatigue, pain, and nausea/vomiting). Additionally, the questionnaire includes several single items assessing specific symptoms. For the five functional scales and the global quality of life scale, higher scores indicate better functioning and quality of life; conversely, for the symptom scales and single symptom items, higher scores reflect greater symptom severity ​[13]​.

### Risk of bias

**Fig. 2:**
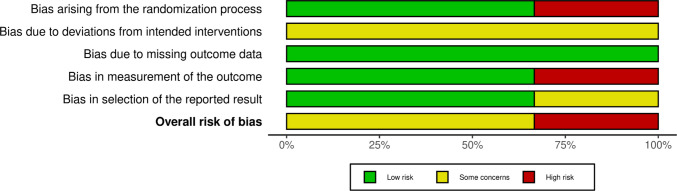
Summary of risk of bias assessed by the Cochrane tool

**Fig. 3:**
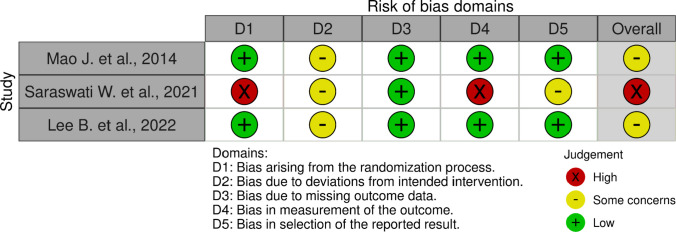
Risk of bias assessed by the Cochrane tool

The three included randomized clinical trials were analyzed using the Rob-2 tool, and after classification, the data were submitted to the Robvis application for the creation of graphs *(*Figures 2 and 3*)* which provide a better visualization of the results. Analyzing the general graph (Figure 2), we identified that domain 1 (D1), which concerns the randomization of the trial, and domain 4 (D4), regarding outcome measurement, were the most problematic among the analyzed studies. The absence of blinding of assessors, the use of non-robust or poorly reported randomization methods, and the subjectivity in outcome measurement are some factors that may have contributed to the "high risk of bias" classification observed in these studies. On the other hand, domains 3 and 5 (D3 and D5), corresponding to missing data and selection of reported results, respectively, showed "low risk" or "some concerns," suggesting that the included studies, for the most part, managed to maintain relatively adequate participant adherence and reported outcomes as described in the pre-established protocols. However, domain 2 (D2) predominantly presented "some concerns," indicating methodological vulnerability regarding fidelity to the proposed intervention and the presence of possible deviations from conduct or absence of blinding of participants and involved professionals, often justified by the nature of the intervention.

In the individual study analysis presented in Figure 3, Mao et al., 2014 obtained an overall classification of "some concerns," because although it presented a generally robust methodology, we noted possible failures in controlling adherence to the intervention or maintaining blinding. The study by Saraswati et al., 2021, on the other hand, presented critical flaws in randomization (D1) and outcome measurement (D4), compromising the reliability of its results. In contrast, the study by Lee et al., 2022 presented a solid methodology, but due to the lack of clear information on controlling intervention fidelity or more robust blinding, we classified it as "some concerns." Despite this, we consider it the most reliable among those included, with the other four domains classified as "low risk of bias."

### Methodological quality of included clinical trials

The methodological quality of the studies included in this review, using the PEDro scale, as shown in Table 3, determined that the trials by Mao et al., 2009 and Zhao et al., 2023 are of low quality, with scores below 6, which is an expected classification as they are not randomized clinical trials. The clinical trials by Mao et al., 2014; Saraswati et al., 2021 and Lee B. et al., 2022 scored 9/10, 6/10, and 8/10, respectively, and were therefore classified as high methodological quality by the PEDro scale. The scale was applied by two independent researchers, and discrepancies were resolved by a third researcher.

**Table 3:** Assessment of methodological quality (PEDro scale)

Author/Year	1	2	3	4	5	6	7	8	9	10	11	Total
Mao et al., 2009	** + **	**-**	**-**	**-**	**-**	**-**	**-**	**-**	**-**	**-**	**-**	0
Mao et al., 2014	** + **	** + **	** + **	** + **	** + **	**-**	** + **	** + **	** + **	** + **	** + **	9
Saraswati et al., 2021	** + **	** + **	**-**	** + **	**-**	**-**	**-**	** + **	** + **	** + **	** + **	6
Lee et al., 2022	** + **	** + **	**-**	** + **	**-**	**-**	** + **	** + **	** + **	** + **	** + **	8
Zhao et al., 2023	** + **	**-**	**-**	** + **	**-**	**-**	**-**	** + **	** + **	** + **	** + **	5

**Legends:** 1 Eligibility criteria, 2 Random allocation, 3 Concealed allocation, 4 Baseline Comparability,

5 Blind subjects, 6 Blind therapists, 7 Blind assessor, 8 < 15% of dropout, 9 Intention to treat analysis, 10 Between groups comparison, 11 Point estimates and variability

### Intervention

A total of 251 patients were included in the clinical trials analyzed in this review, of whom 132 were treated with EA. The frequency of treatment varied little between studies, with most treatments performed 2 to 3 times per week for 20 to 30 minutes. The duration of treatment was 4 to 8 weeks; however, the study by Zhao et al., 2023 was based on each patient's chemotherapy cycles and did not report the duration of the study in weeks. Both the frequency and duration of treatment appeared to have no impact on the results. The selection of acupuncture points varied across studies and was tailored to the signs and symptoms of the patient population. Among the points employed - ST36, SP6, LI4, GV20, EX-HN3, HT7, PC6, BL63, KI4, ST36, SP6, and LR3 - Zusanli (ST36), Sanyinjiao (SP6), and Hegu (LI4) were most consistently associated with fatigue management. The frequency of electrical stimulation applied through the needles ranged widely from 0.2 Hz to 25 Hz across trials, but these variations did not appear to influence the therapeutic effect of EA. In studies that included a Simulated Electroacupuncture (SEA) control group, needles were placed superficially without penetrating the skin, positioned at non-acupuncture points, and connected to inactive devices that mimicked active treatment by emitting identical sounds and lights without delivering electrical current.

### Fatigue assessed by specific instruments

Mao et al., 2009, in a pilot study of 12 postmenopausal women with breast cancer receiving aromatase inhibitors, reported a significant reduction in fatigue measured by the BFI, from 4.4/10 (±2.2) to 1.9/10 (±2.0) after eight weeks of treatment (p = 0.005), along with improvements in pain, joint stiffness, and anxiety. Building on these findings, the same group conducted a three-arm randomized clinical trial in 2014 (EA, simulated EA [SEA], and usual care control) including 67 women with AI-induced arthralgia. Intra-group analyses revealed that the EA group demonstrated progressive and clinically meaningful reductions in fatigue scores as measured by the BFI: −0.4 (95% CI −1.6 to 0.7) at week 4, −1.4 (95% CI −2.7 to −0.1) at week 8, and −1.4 (95% CI −2.7 to −0.1) at week 12, indicating sustained improvement over time. In contrast, the SEA and control groups did not exhibit consistent statistically significant changes during these periods (global *p* for EA vs. control = 0.0095; SEA vs. control = 0.18). In baseline-adjusted inter-group analyses, EA produced significantly greater improvements in fatigue compared with control group at 8 weeks (mean difference −2.0; 95% CI −3.4 to −0.5; *p* = 0.0034) and 12 weeks (−1.6; 95% CI −3.2 to −0.07; *p* = 0.022). In turn, SEA did not differ significantly from the control group in fatigue.

Lee et al., 2022 evaluated EA primarily for cancer-related insomnia but included fatigue, assessed using the FACT-F, as a secondary outcome and was therefore considered in this review. This randomized three-arm trial (EA, EAS, and usual care) achieved high adherence (95.45%). Intra-group analyses demonstrated that the EA group, the mean FACT-F score increased from 107.88/160 (95% CI 96.87–118.88) at baseline to 120.00/160 (95% CI 103.35–136.65) at 4 weeks post-treatment, approaching but not reaching statistical significance (within-group *p* = 0.0579). The EAS group exhibited a mean change from 88.50/160 (95% CI 75.25–101.75) at baseline to 94.58/160 (95% CI 81.44–107.72) at 4 weeks, which was not statistically significant (within-group *p* = 0.2445). Similarly, the control group demonstrated a modest increase from 99.88/160 (95% CI 82.21–117.54) at baseline to 106.63/160 (95% CI 82.76–130.49) at 4 weeks, without statistical significance (within-group *p* = 0.4331). In the inter-group comparisons at 4 weeks, a statistically significant difference was observed between electroacupuncture and sham (between-group *p* = 0.0305), whereas no significant difference was found between electroacupuncture and usual care (between-group *p* = 0.3111).

More recently, Zhao et al., 2023 investigated EA for post-adjuvant chemotherapy fatigue in breast cancer. Although designed as a non-randomized trial, the study introduced a novel approach by exploring genetic polymorphisms (SNPs) associated with therapeutic response. Although the study does not provide detailed descriptive statistics (e.g., means and standard deviations), it reports clinically and statistically relevant findings regarding fatigue based on the BFI, allowing both intra-group and inter-group interpretation. In the EA group, intra-group analysis showed that 63 patients (82.9%) presented with moderate to severe fatigue (BFI > 3) at baseline, which decreased to 46 patients (60.5%) after treatment. This represents a 26.9% absolute reduction in the proportion of patients with clinically significant fatigue, and this improvement was statistically significant (*p* < 0.05), indicating a meaningful within-group reduction in fatigue severity following EA. In contrast, in the control group, 40 of 48 patients exhibited BFI > 3 at baseline, and 36 patients remained above this threshold after the same observation period, reflecting only a modest reduction that was not reported as statistically significant within the group. In the inter-group comparison, the magnitude of fatigue relief differed significantly between the EA and control groups (*p* = 0.037), demonstrating that the reduction in moderate to severe fatigue was significantly greater among patients receiving EA than among those who did not receive the intervention. Collectively, these findings support both a significant intra-group improvement in the EA group and a superior inter-group effect compared with control.

### Fatigue assessed by non-specific instruments

Saraswati et al., 2021 investigated EA in a different clinical context, enrolling patients with stage III cervical cancer undergoing conventional pain management. Although analgesia was the primary endpoint, fatigue was assessed using the Indonesian version of the EORTC QLQ-C30 and showed significant improvement in the EA group. Intra-group analysis demonstrated that the EA group showed a statistically significant reduction in fatigue scores from a pre-test median of 72.17/100 (33.33–89.00) to a post-test median of 44.33/100 (41.67–66.67), with a within-group significance (p = 0.001). In contrast, the control group did not exhibit improvement; fatigue scores remained unchanged, with a pre-test median of 66.67 (33.33–77.67) and a post-test median of 66.67 (33.33–77.67), although a statistically significant within-group value was reported (p = 0.038), likely reflecting statistical variation rather than clinical improvement given the identical central values. In the inter-group comparison, the difference in fatigue reduction between the treatment and control groups was statistically significant (p = 0.012), indicating that electroacupuncture produced a significantly greater reduction in fatigue compared with control. These findings support both a robust intra-group improvement in fatigue in the electroacupuncture group and a superior inter-group effect relative to usual care. An interesting relationship that this study establishes is that improvements in the EA group were accompanied by a mean increase of 88.57 ± 52.46 pg/ml in plasma β-endorphin levels, compared with only 12.86 ± 56.76 pg/ml in the control group (p = 0.001).

### Safety and adverse effects

In the studies by Mao et al., 2009; Mao et al., 2014 and Saraswati et al., 2021 no adverse effects related to EA were reported. Zhao et al., 2023 described incidences of adverse events, including bleeding at the needle insertion site (3.9%), hematoma at the insertion site (2.6%), pain during acupuncture (1.3%), inflammation at the insertion site (2.6%), dizziness (1.3%), and palpitations (1.3%). These events were considered limited and tolerable. Lee et al., 2022 described 23 adverse events over 185 visits, with no significant between-group difference in the incidence of adverse events (10 cases/96 visits [10.42%] in the EA group, including 2 cases of headache and 1 case each of cough, lumbago, common cold, enteritis, dizziness, knee joint pain, rhinitis, and dyspepsia; 7 cases/57 visits [12.28%] in the SEA group, including 2 cases of common cold and 1 case each of shoulder joint pain, skin allergy, lymphadenitis, hematuria, and dyspepsia; and 6 cases/32 visits [18.75%] in the usual care group, including 1 case each of arthritis, skin spot, diarrhea, dyspepsia, toothache, and ileus; Fisher’s exact test, P = 0.4323). One participant in the control group was hospitalized for 10 days due to postoperative ileus during the trial period, but the authors considered them not relevant to the study. Overall, all reported adverse events were deemed unrelated to the intervention and resolved spontaneously.

## Discussion

### Possible mechanisms of action

One possible explanation for the effect of EA in reducing CRF is its ability to modulate inflammatory responses by significantly lowering serum levels of inflammatory markers such as interleukin-6 (IL-6) and tumor necrosis factor-alpha (TNF-α), which are typically elevated in patients with CRF [9]. At the cellular level, EA may exert protective effects on mitochondrial function and promote ATP synthesis, thereby mitigating oxidative stress. Additionally, it appears to modulate intracellular signaling pathways that influence cellular homeostasis and energy metabolism, potentially leading to a reduction in the production of pro-inflammatory cytokines.. possible mechanism involves regulation of the gut–microbiota–brain axis. Stimulation of specific acupoints such as ST36 and SP6 - employed in the clinical trials of Zhao et al., 2023 and Saraswati et al., 2021 - may alter the composition of the intestinal microbiota by increasing beneficial bacterial populations, promoting the production of neuroactive metabolites, and modulating systemic inflammatory responses [10, 14]. From a neuroendocrine perspective, chronic hyperactivity of the hypothalamic–pituitary–adrenal (HPA) axis is associated with exhaustion, sleep disturbances, and heightened fatigue perception. EA has demonstrated the capacity to attenuate HPA axis hyperactivity by reducing cortisol secretion and improving autonomic regulation. Furthermore, it may enhance gamma-aminobutyric acid (GABA) levels and receptor expression, as well as stimulate melatonin secretion, thereby contributing to improved sleep quality and a reduction in CRF [10]. EA exerts differential effects depending on stimulation intensity. At low intensity, it activates the vagal–adrenal axis, whereas at high intensity, it stimulates the sympathetic–splenic axis. This leads to the release of catecholamines into the bloodstream or the spleen. These substances influence both immune cells and cancer cells, potentially exerting either anti-tumor or pro-tumor effects depending on the specific context. An important finding is that EA at the Zusanli (ST36) acupoint can induce targeted catecholamine release, thereby directly modulating immune cell function [15–17].

Although these mechanisms suggest a multifactorial and potentially powerful role of EA in the management of CRF, significant gaps remain in the current body of evidence, rendering these mechanistic pathways largely speculative.

### Limitations of the included studies

Despite growing interest in the use of EA as a complementary strategy for managing CRF, there remains a substantial lack of robust studies with representative samples and methodologically rigorous designs capable of supporting definitive conclusions regarding its efficacy.

Analyzing the studies included in this review, the pilot trial by Mao et al., 2009, although relevant, lacked a control group, preventing assessment of the specific effect of EA beyond placebo. Moreover, its small and homogeneous sample limited generalizability. Nevertheless, as a feasibility study, it provided a valuable foundation for subsequent trials, suggesting a potential role of EA in modulating multiple symptoms, possibly through neuroendocrine and endogenous opioid pathways. In the 2014 trial by the same authors, the three-arm randomized design with placebo control and more robust analyses represented a methodological improvement. However, limitations persisted, including a modest sample size, potential placebo effects (given that the EAS group showed improvements in depression), and a short follow-up period of only four weeks post-intervention, which precluded conclusions about long-term efficacy. The study by Saraswati et al., 2021 also presented constraints, such as the absence of a placebo/EAS group, a brief duration (three weeks), and a small sample size (n = 28), all of which limit the ability to distinguish specific effects of EA and to assess the persistence of benefits. Lee et al., 2022, despite employing a high-quality methodology, faced recruitment challenges, resulting in a very small sample (n = 22) and reduced statistical power to detect differences in secondary outcomes such as fatigue. Finally, Zhao et al., 2023 conducted an innovative single-arm trial incorporating genetic biomarkers to explore predictors of response. Although this study included a comparatively larger sample, it lacked an EAS group and participant blinding, raising the risk of overestimation of effects. Still, its integration of molecular data represented a meaningful advance in understanding the mechanisms of EA in CRF.

The assessment of CRF may vary substantially depending on the instrument employed, particularly when comparing fatigue-specific scales - such as the BFI and the FACT-F, which are designed specifically to evaluate fatigue and allow for greater sensitivity in measuring symptom severity - with multidimensional quality-of-life instruments, such as the EORTC QLQ-C30. The latter assesses fatigue as one of several domains within a broader quality-of-life questionnaire that also encompasses physical, emotional, and social dimensions. While this approach provides a comprehensive overview of the patient’s overall condition, it offers less depth in the isolated evaluation of fatigue. This methodological distinction may have directly influenced the outcomes of the clinical trials, as fatigue-specific instruments generally demonstrate greater responsiveness and statistical power to detect the effects of interventions targeting fatigue, whereas global instruments may dilute such changes across multiple concurrently assessed domains.

Finally, in four of the five included studies, fatigue was not defined as the primary outcome. Consequently, sample size calculations were typically not powered to detect statistically significant differences in this specific symptom, thereby reducing the studies’ ability to identify true intervention effects. Moreover, the therapeutic protocols may not have been primarily structured with fatigue as the central focus; thus, even if a clinical benefit exists, it may have been underestimated. Therefore, the classification of fatigue as a secondary outcome limits the interpretability of the findings and weakens conclusions regarding the true efficacy of electroacupuncture in this context.

### Limitations of this review

This study presents important limitations that affect the robustness and strength of its conclusions. A limited number of randomized controlled trials were included, which restricts the representativeness of the findings and reduces the inferential power regarding the efficacy of the intervention. Furthermore, the absence of a quantitative synthesis (meta-analysis), due to substantial clinical and methodological heterogeneity among the included studies, precluded estimation of a pooled effect size and a more precise statistical evaluation of the results. The analyzed population was heterogeneous with respect to cancer type, disease stage, phase of oncological treatment, and participants’ clinical characteristics, thereby compromising comparability across studies. Additionally, there was considerable variability in intervention protocols, including differences in technical parameters, frequency, duration, and number of sessions, as well as the use of distinct outcome assessment instruments, particularly for fatigue. This extensive clinical and methodological heterogeneity hindered data synthesis, limited the possibility of standardizing the evidence, and reduced the overall methodological quality of this systematic review, resulting in more cautious and less generalizable conclusions. We chose not to include other interventions in this review (such as traditional manual acupuncture or acupressure) in order to provide greater visibility to EA in clinical practice. Given that EA is a relatively recent technique with potentially greater promise, due to its higher reproducibility, objectivity, and quantifiability compared to the aforementioned techniques, which, despite having strong clinical and scientific evidence, depend entirely on the manual manipulation skills of the acupuncturist.

### Recommendations

To rigorously investigate the effects of EA, a well-designed Randomized Controlled Trial with strong methodological standards and adequate statistical power is recommended. The study should include an a priori sample size calculation based on fatigue as the primary outcome, proper randomization with allocation concealment, and, ideally, blinding of participants and outcome assessors through the use of a placebo group (e.g., sham electroacupuncture with inactive stimulation or subtherapeutic parameters). Furthermore, a three-arm design (electroacupuncture, manual acupuncture, and placebo) would allow differentiation between the specific effects of electrical stimulation and those attributable to needle insertion and contextual or nonspecific effects of the intervention. Rigorous standardization of acupoints, frequency, intensity, session duration, and number of sessions is essential to minimize heterogeneity, as is the use of validated, fatigue-specific instruments as the primary outcome measure. Medium- and long-term follow-up, intention-to-treat analysis, and transparent reporting are also recommended. Given that the evidence supporting manual acupuncture is more established in the scientific literature, whereas EA is a more recent technique, high-quality methodological studies are crucial to distinguish mechanisms of action, magnitude of effect, and clinical applicability of each approach.

## Conclusion

The included studies present methodological limitations, high risk of bias, heterogeneous intervention protocols (including variability in acupoints, frequency, intensity, and duration), non-standardized fatigue assessment tools, small sample sizes, and short follow-up periods, all of which hinder direct comparisons and the development of standardized clinical protocols. Additionally, differences in cancer type, disease stage, and treatment phase limit the generalizability of the findings. Although preliminary evidence indicates that electroacupuncture may be a promising therapeutic approach for cancer-related fatigue, current clinical evidence remains insufficient to support strong, universal recommendations, highlighting the need for rigorously designed randomized controlled trials with larger samples, standardized protocols, long-term follow-up, and clinically relevant outcomes.

## Data Availability

No datasets were generated or analysed during the current study.

## References

[1] Global cancer burden growing, amidst mounting need for services. Available from: https://www.who.int/news/item/01-02-2024-global-cancer-burden-growing--amidst-mounting-need-for-services. Accessed 13 June 2025 PMC11115397 38438207

[2] Câncer - OPAS/OMS | Organização Pan-Americana da Saúde. Available from: https://www.paho.org/pt/topicos/cancer. Accessed 13 June 2025

[3] Cancer Diagnosis and Treatment | Cancer Survivors | CDC. Available from: https://www.cdc.gov/cancer-survivors/patients/index.html. Accessed 13 June 2025

[4] Huynh TTM, Falk RS, Hellebust TP, Dale E, Astrup GL, Hjermstad MJ, et al (2024) Chronic fatigue in long-term survivors of head and neck cancer treated with radiotherapy. Radiotherapy Oncol 1 195. Available from: https://pubmed.ncbi.nlm.nih.gov/38518958/. Accessed 21 June 2025 10.1016/j.radonc.2024.110231 38518958

[5] Mota DDC de F, Pimenta CA de M (2002) Fadiga em pacientes com câncer avançado: conceito, avaliação e intervenção. Revista Brasileira de Cancerologia. 48(4):577–83. Available from: https://rbc.inca.gov.br/index.php/revista/article/view/2172. Accessed 21 June 2025

[6] Al Maqbali M, Al Sinani M, Al Naamani Z, Al Badi K, Tanash MI (2021) Prevalence of Fatigue in Patients With Cancer: A Systematic Review and Meta-Analysis. J Pain Symptom Manage 61(1):167–189.e14. Available from: https://pubmed.ncbi.nlm.nih.gov/32768552/. Accessed 19 June 2025 10.1016/j.jpainsymman.2020.07.037 32768552

[7] Traditional Chinese Medicine: What You Need To Know | NCCIH. Available from: https://www.nccih.nih.gov/health/traditional-chinese-medicine-what-you-need-to-know. Accessed 20 June 2025

[8] Xie L, Ng DQ, Heshmatipour M, Acharya M, Coluzzi P, Guerrero N, et al (2023) Electroacupuncture for the management of symptom clusters in cancer patients and survivors (EAST). BMC Complement Med Ther 23(1). Available from: https://pubmed.ncbi.nlm.nih.gov/36973688/. Accessed 25 June 2025 10.1186/s12906-023-03926-9 PMC10041509 36973688

[9] Qing P, Zhao JF, Zhao CH, Hu J, Lin YL, He KJ (2020) [Effect of acupuncture on patients with cancer-related fatigue and serum levels of CRP, IL-6, TNF-α and sTNF-R1]. Zhongguo Zhen Jiu 40(5):505–9. Available from: https://www.ncbi.nlm.nih.gov/pubmed/32394658. Accessed 21 June 2025 10.13703/j.0255-2930.20190423-k0002 32394658

[10] Yang X, Li G, Yang J, Ma X (2024) Mechanism and application of acupuncture and electro-acupuncture associated with cancer therapy. MedComm - Oncol 1;3(2)

[11] Mendoza TR, Wang XS, Cleeland CS, Morrissey M, Johnson BA, Wendt JK, et al (1999) The rapid assessment of fatigue severity in cancer patients. Cancer 85(5):1186–96. Available from: https://pubmed.ncbi.nlm.nih.gov/10091805/. Accessed 21 June 2025 10.1002/(sici)1097-0142(19990301)85:5<1186::aid-cncr24>3.0.co;2-n 10091805

[12] Ishikawa NM, Claudio L, Thuler S, Giglio AG, Serodio Da Rocha Baldotto C, Coelho De Andrade CJ, et al (2008) Reproducibility of Functional Assessment of Cancer Therapy- Fatigue (FACT-F) Questionnaire for Cancer Patients Available from: https://ninho.inca.gov.br/jspui/handle/123456789/4852. Accessed 3 July 2025

[13] Kaasa S, Bjordal ’ K, Aaronson N, Moum T, Wist E, Hagen S, et al (1995) Original Paper The EORTC Core Quality of Life Questionnaire (QLQX30): Validity and Reliability When Analysed With Patients Treated With Palliative Radiotherapy. EurJ Cancer 31:226–2263. Accessed 2 July 2025 10.1016/0959-8049(95)00296-0 8652253

[14] Lv Z, Gu YM, Liu RD, Su KQ, Ruan X Di, Chang YN, et al (2022) The Clinical Observation and Mechanism of Acupuncture on Cancer-Related Fatigue of Breast Cancer Based on “Gut-Brain Axis”: Study Protocol for a Randomized Controlled Trial. Dis Markers 2022(1):8099595. Available from: 10.1155/2022/8099595. Accessed 3 July 2025 10.1155/2022/8099595 PMC9107368 35578688

[15] Liu S, Wang Z, Su Y, Qi L, Yang W, Fu M, et al (2021) A neuroanatomical basis for electroacupuncture to drive the vagal-adrenal axis. Nature 598(7882):641. Available from: https://pmc.ncbi.nlm.nih.gov/articles/PMC9178665/. Accessed 21 June 2025 10.1038/s41586-021-04001-4 PMC9178665 34646018

[16] Liu S, Wang ZF, Su YS, Ray RS, Jing XH, Wang YQ, et al (2020) Somatotopic organization and intensity dependence in driving distinct NPY-expressing sympathetic pathways by electroacupuncture. Neuron. 108(3):436. Available from: https://pmc.ncbi.nlm.nih.gov/articles/PMC7666081/. Accessed 5 July 2025 10.1016/j.neuron.2020.07.015 PMC7666081 32791039

[17] Wackerhage H, Christensen JF, Ilmer M, von Luettichau I, Renz BW, Schönfelder M (2022) Cancer catecholamine conundrum. Trends Cancer. 8(2) 110–22. Available from: https://www.cell.com/action/showFullText?pii=S2405803321002107. Accessed 5 July 2025 10.1016/j.trecan.2021.10.005 34776398

[18] Haddaway NR, Page MJ, Pritchard CC, McGuinness LA (2022) PRISMA2020: An R package and Shiny app for producing PRISMA 2020-compliant flow diagrams, with interactivity for optimised digital transparency and Open Synthesis. Campbell Syst Rev 18(2):e1230. Available from: /doi/pdf/10.1002/cl2.1230. Accessed 15 July 2025 10.1002/cl2.1230 PMC8958186 36911350

[19] Mao JJ, Bruner DW, Stricker C, Farrar JT, Xie SX, Bowman MA, et al (2009) Feasibility Trial of Electroacupuncture for Aromatase Inhibitor—Related Arthralgia in Breast Cancer Survivors. Integrative Cancer Therapies. 8(2):123–9. Available from: https://pubmed.ncbi.nlm.nih.gov/19679620/. Accessed 7 July 2025 10.1177/1534735409332903 PMC3569528 19679620

[20] Mao JJ, Farrar JT, Bruner D, Zee J, Bowman M, Seluzicki C, et al (2014) Electroacupuncture for fatigue, sleep, and psychological distress in breast cancer patients with aromatase inhibitor‐related arthralgia: A randomized trial. Cancer 120(23):3744–51. Available from: https://pmc.ncbi.nlm.nih.gov/articles/PMC4239308/. Accessed 5 July 2025 10.1002/cncr.28917 PMC4239308 25077452

[21] Saraswati W, Wardani R, Suhatno S, Hartono P, Imandiri A (2021) The effect of electroacupuncture therapy on pain, plasma β-endorphin, and quality of life of stage III cervical cancer patients: a randomized control trial. J Acupunct Meridian Stud 14(1):4–12. 10.51507/j.jams.2021.14.1.4. Accessed 7 June 2025 35770596 10.51507/j.jams.2021.14.1.4

[22] Lee B, Kim BK, Kim M, Kim AR, Park HJ, Kwon OJ et al (2022) Electroacupuncture for treating cancer-related insomnia: a multicenter, assessor-blinded, randomized controlled, pilot clinical trial. Bmc Complementary Medicine Therapies. 22(1):77. Available from: https://pubmed.ncbi.nlm.nih.gov/35303841/. Accessed 5 July 2025 10.1186/s12906-022-03561-w PMC8932204 35303841

[23] Zhao F, Shen G, Ren D, Wang M, Liu Z, Zhao Y et al (2023) Screening single nucleotide polymorphisms predicting the efficacy of electroacupuncture for fatigue treatment in patients with breast cancer following adjuvant chemotherapy. Biochem Genet 62(2):1291–1303. 10.1007/s10528-023-10477-8 37596508 10.1007/s10528-023-10477-8

